# Prevalence and correlates of short and long sleep duration among majority-Hispanic young adult survivors of childhood cancer

**DOI:** 10.1007/s11764-025-01949-4

**Published:** 2025-12-12

**Authors:** Marcie D. Haydon, Julia Stal, Sunmin Lee, Kimberly A. Miller, Wendy Cozen, David R. Freyer, Joel Milam

**Affiliations:** 1 Department of Population Health and Disease Prevention, Joe C. Wen School of Population and Public Health, University of California Irvine, Irvine, CA USA; 2 Department of Medical Oncology, Dana-Farber Cancer Institute, Boston, MA USA; 3 Department of Epidemiology and Biostatistics, Joe C. Wen School of Population and Public Health, University of California Irvine, Irvine, CA USA; 4 Chao Family Comprehensive Cancer Center, University of California Irvine, Orange, CA USA; 5 Division of Hematology/Oncology, Department of Medicine, School of Medicine, University of California Irvine, Orange, CA USA; 6 Department of Public Health and Population Science, Keck School of Medicine, University of Southern California, Los Angeles, CA USA; 7 Department of Pathology, School of Medicine, University of California Irvine, Orange, CA USA; 8 Department of Pediatrics, Keck School of Medicine, University of Southern California, Los Angeles, CA USA; 9 Department of Medicine, Keck School of Medicine, University of Southern California, Los Angeles, CA USA

**Keywords:** Childhood cancer survivors, Sleep, Sleep duration, Cancer survivorship

## Abstract

**Purpose:**

Both short (< 7 h) and long (> 9 h) sleep duration are associated with poor health. Yet, little is known about sleep duration patterns and correlates in young adult childhood cancer survivors (CCS).

**Methods:**

Data were from Project Forward, a population-based survey of young adult CCS in Los Angeles County. Average sleep duration, demographic and medical characteristics (e.g., treatment intensity), and mental (i.e., well-being, depressive symptoms) and physical (i.e., general health, comorbidities) health were measured via self-report. Chi-square and one-way ANOVAs assessed correlations between characteristics and short and long sleep duration (versus recommended; 7–9 h). Multivariable linear and negative binomial regressions examined relationships between sleep duration and mental and physical health.

**Results:**

Participants (*N* = 1,062, Mean age = 26.2 years, 50% female, 51% Hispanic) were, on average, 14.5 years (range: 5–22) post-diagnosis. Overall, 30% reported short and 7% reported long sleep. Short sleep was associated with older age, being employed, and having children (*p*s ≤ .001). Unemployment and lower educational attainment were associated with long sleep (*p*s ≤ .001). Both short and long sleep were associated with lower well-being (*p*s ≤ .001), greater depressive symptoms (*p*s ≤ .001), poorer self-rated general health (*ps* ≤ .012), and more comorbid conditions (*ps* = .001), compared to recommended sleep duration. Associations with physical health remained largely significant controlling for depressive symptoms (*p*s < .025).

**Conclusions:**

We found unique correlates of short and long sleep duration and associations with poorer mental and physical health among a large, diverse sample of young adult CCS.

**Implications for Cancer Survivors:**

As a modifiable behavior, sleep duration is a relevant intervention target for improving outcomes after cancer.

Sleep duration is an underrecognized health behavior in cancer survivorship, particularly among young cancer survivors. In the general population, both short (< 6 or 7 h) and long (≥ 9 or 10 h) sleep duration are associated with poor health and may confer risk for chronic health conditions, including depression, diabetes, and cardiovascular disease [1–4]. Inadequate sleep may alter immune function and the autonomic nervous system [5–7], potentially contributing to these associations. Notably, several large studies have also demonstrated variability in sleep duration by race/ethnicity, with minoritized populations reporting more short or long sleep [8, 9]. Among patients and survivors of adult-onset cancer, sleep duration is linked to increased all-cause and cancer-specific mortality—though these associations differ by cancer type and stage [10, 11]—and has been shown to adversely affect treatment-induced physical symptoms, including cognitive impairment [12] and fatigue [13]. Distressingly, sleep disturbances can persist for years following curative cancer treatment and significantly impair quality of life [14].

Little is known about the relationship between sleep duration and health outcomes in childhood cancer survivors (CCS), despite this group being at high risk for treatment-related late effects, including chronic health conditions [15, 16], persistent physical symptoms [17, 18], and distress [19, 20]. Only a few studies, to date, have examined sleep duration among CCS. Reports from the Childhood Cancer Survivor Study (*n* = 1,933) and the St. Jude Lifetime Cohort (*n* = 911) suggest that 35–44% of adult CCS report short sleep, and very few (1%) report long sleep [21, 22]. Among CCS, short sleep was associated with an increased risk of depression, anxiety, and chronic conditions of the pulmonary, endocrine, and gastrointestinal systems relative to recommended sleep duration [21]. However, low endorsement of long sleep prevented examination of correlates, and participants in these studies were primarily non-Hispanic white (≥ 84%). To date, information on sleep duration in racial/ethnic minoritized CCS is lacking. Notably, getting adequate sleep may ameliorate loss of vitality and function in CCS [23]; yet, historically, sleep health has received less attention than other health behaviors (e.g., healthy eating, physical activity) among this group.

Here, we sought to characterize sleep duration in a diverse, population-based sample of young adult CCS (51% Hispanic). Our aims were to (1) examine the prevalence of short (< 7 h) and long (≥ 9 h) sleep duration (as defined by the National Sleep Foundation [24]), (2) identify demographic (i.e., age, gender, ethnicity, parent status, employment, education) and medical (i.e., type of cancer diagnosis, age at diagnosis, treatment intensity) correlates of both short and long sleep, and (3) assess relationships between sleep duration and self-reported mental (i.e., well-being, depressive symptoms) and physical (i.e., general health, number of comorbid conditions) health outcomes.

## Methods

### Participants and procedures

Data were from the Project Forward Cohort, a cross-sectional, population-based study that examined risk and protective factors of cancer-related follow-up care among young adult CCS [25]. Participants were identified through the Los Angeles Cancer Surveillance Program—part of the national Surveillance, Epidemiology, and End Results (SEER) program—and were (1) 18 to 39 years old at study entry, (2) diagnosed with any cancer type (stage I + for brain, stage II + for others) at 0 to 19 years of age in Los Angeles County between the years 1996 and 2010, and (3) at least 5-years post-diagnosis. Recruitment methods included introductory postcards and self-report survey mailings in English and Spanish. Participants responded to the survey at one timepoint through the internet, mail, or phone. Study participants received $20 cash upon survey completion and a lottery entry ($300). Additional study details are described elsewhere [25]. The University of Southern California Institutional Review Board, the California State Committee for the Protection of Human Subjects, and the California Cancer Registry approved all study procedures.

### Measures

**Demographic, Medical, and Treatment Characteristics.** Age at cancer diagnosis, interval since diagnosis, age at questionnaire, biological sex, race/ethnicity (non-Hispanic white, Hispanic, Asian, Black, and other/unknown), quintiles of neighborhood socioeconomic status (nSES) at diagnosis, and cancer type were obtained from the cancer registry. nSES is a census-based multi-component composite score [26, 27]; quintiles are relative to California’s statewide distribution, with 1 representing the lowest quintile and 5 representing the highest quintile of nSES. Information on employment, education, relationship status, parental status, and cancer relapse/recurrence was collected via self-report. Treatment intensity was calculated using both registry and self-report variables as described previously [25, 28].

**Sleep Characteristics**. Sleep duration was assessed using one self-report item, “On average, how many hours of sleep do you get in a 24-hour period?,” which has been used previously in studies looking at sleep duration and health among young adults [29, 30]. Sleep disturbance within the past week was assessed using one item from the Center for Epidemiological Studies Depression Scale (CES-D), “My sleep was restless” (4-point Likert scale; 0= *Rarely or none of the time [less than one day]* to 3 = *Most or all of the time [5–7 days]*) [31].

**Psychological Well-being.** Psychological well-being was measured using the 14-item Mental Health Continuum-Short Form (MHC-SF) [32]. The MHC-SF measures three components of well-being: hedonic, eudaimonic social, and eudaimonic psychological well-being. Responses pertained to the previous month and were scored on a 6-point Likert scale (0 = *never*, 5 = *every day*), with higher scores indicating greater well-being.

**Depressive Symptoms.** Depressive symptoms were measured using 13 items from the CES-D [31]. The 7-item somatic subscale [33]—which includes items on sleep and fatigue—was excluded to prevent conceptual overlap. Participants indicated how often they experienced symptoms (e.g., feelings of loneliness, guilt, worthlessness, depressed mood) during the previous week on a 4-point Likert scale (0 = *Rarely or none of the time (less than one day)*, 3 = *Most or all of the time (5–7 days)*). Items were summed to create a total score, with higher scores representing higher depressive symptoms.

**Physical Health.** Self-rated general health was assessed using one item from the RAND 36-item Short Form Health Survey (SF-36), “How would you rate your general health overall?” (5-point Likert scale; 0= *Poor*, 4 = *Excellent*) [34]. Comorbidities were assessed by an 11-item checklist (yes/no) comprised of common late health effects attributable to cancer diagnosis and treatment, namely an inability to have children, heart problems, secondary neoplasm, weight gain, liver damage, hearing problems, difficulties with learning and memory, difficulty breathing or lung problems, poor eyesight, problems with sexual functioning, and frequent bone fractures. The checklist items were summed to create an overall comorbidity score.

### Analytic strategy

Participants were stratified based on self-reported sleep duration—short (< 7 h), recommended (7–9 h), or long (> 9 h) sleep—following the National Sleep Foundation’s recommendations for young adults [24]. Individuals who indicated exceptionally short (< 4 h; *n* = 4) or long (> 14 h; *n* = 6) sleep duration were excluded, as these values may be unreliable or indicate abnormally significant disease or distress. Those who did not provide sleep data (*n* = 31) were also excluded. Differences between short and long sleep duration and recommended sleep duration on demographic and medical characteristics and restless sleep were assessed using one-way analysis of variance (ANOVA), Chi-square tests, and Fisher’s exact tests. Associations between short and long sleep duration, versus recommended sleep duration, and mental and physical health were measured in separate models using multivariable linear regressions (dependent variables: well-being, depressive symptoms, self-rated general health) and negative binomial regression (dependent variable: number of comorbidities) controlling for age at survey completion, sex, race/ethnicity, and treatment intensity. Given the bidirectional relationships between sleep, depressive symptoms, and physical health (e.g., [35–37]), physical health outcomes were re-analyzed to control for depressive symptoms. Post hoc effect modification analysis was also conducted to examine whether associations between short and long sleep duration and mental and physical health differed by Hispanic ethnicity (vs. non-Hispanic white). Other racial/ethnic groups were excluded due to small cell sizes. Statistical significance was determined as a probability value (*p*-value) less than 0.05 for 2-sided hypothesis tests. All analyses were conducted using the Stata/BE 17 software package.

## Results

### Characteristics of study participants

Demographic, medical, and sleep characteristics are reported in Table 1. On average, participants (*N* = 1,062) were 26.2 years of age, female (50.1%), Hispanic (51.1%), employed full- or part-time (66.7%), and single (69.0%). The average age at diagnosis was 11.5 years old, and the average time from diagnosis was 14.5 years. The most frequently reported cancer types were leukemia (35.5%) and lymphoma (22.1%). Participants reported an average of 7.3 h of sleep per night. Most indicated sleeping 7 to 9 h a night (62.9%); 30% reported short sleep duration (< 7 h), and 7% reported long sleep duration (> 9 h).

**Table 1:** Participant characteristics by sleep duration

	**Total** (*N* = 1,062)	**Short Sleep** **(< 7 h)** (*n* = 322)	**Recommended Sleep (7–9 h)** (*n* = 667)	**Long Sleep** **(> 9 h)** (*n* = 73)	***Test Statistic***
**Column Names**		**[A]**	**[B]**	**[C]**	
**Demographic Characteristics**					
Age at cancer diagnosis					*F*(2, 1059) = 3.12, ***p* = 0.045**^a^
Mean (*SD*)	11.5 (5.3)	12.2 (5.2)	11.5 (5.4)	10.6 (5.0)	
Median	13	13	12	11	
Range	0–19	0–19	0–19	0–19	
Years since diagnosis					*F*(2, 1059) = 2.25, *p* = 0.10
Mean (*SD*)	14.5 (4.4)	14.9 (4.3)	14.4 (4.4)	14.1 (4.4)	
Median	15	15.5	15	14	
Range	5–22	5–22	5–22	6–21	
Age at survey completion					*F*(2, 1059) = 10.60, ***p* < 0.001**^b^
Mean (*SD*)	26.2 (4.9)	27.2 (5.1) **[B]**	25.9 (4.8) **[A]**	24.7 (4.1)	
Median	26	27	25	24	
Range	18–39	18–39	18–39	19–39	
Biological sex, No. (%)					χ^2^ (2) = 4.42, *p* = 0.11
Male	531 (49.9)	168 (51.9)	335 (50.2)	28 (38.4)	
Female	532 (50.1)	155 (48.1)	332 (49.8)	45 (61.6)	
Race or ethnicity, No. (%)					χ^2^ (8) = 9.45, *p* = 0.23
Non-Hispanic white	353 (33.2)	96 (29.8)	237 (35.5)	20 (27.4)	
Hispanic	543 (51.1)	170 (52.8)	335 (50.2)	38 (52.1)	
Asian or Pacific Islander	102 (9.6)	32 (9.9)	63 (9.5)	7 (9.6)	
Non-Hispanic Black	52 (4.9)	20 (6.2)	26 (3.9)	6 (8.2)	
Other or unknown	12 (1.1)	4 (1.2)	6 (0.9)	2 (2.7)	
Employment, No. (%)					χ^2^ (2) = 27.53, ***p* < 0.001**^c^
Employed full- or part-time	704 (66.7)	235 (73.2) **[B]**	440 (66.3) **[A]**	29 (40.9)	
Unemployed	352 (33.3)	86 (26.8)	224 (33.7) **[C]**	42 (59.2) **[B]**	
Student, No. (%)	331 (31.2)	95 (29.5)	211 (31.6)	25 (34.3)	χ^2^ (2) = 0.81, *p* = 0.67
Education, No. (%)					χ^2^ (2) = 12.87, ***p* = 0.001**^d,e^
< College	748 (70.6)	224 (69.8)	459 (69.0)	65 (89.0)	
College Degree	311 (29.4)	97 (30.2)	206 (31.0) **[C]**	8 (11.0) **[B]**	
Neighborhood SES, No. (%)^f^					χ^2^ (8) = 6.65, p = 0.58
Lowest	330 (31.1)	103 (32.0)	205 (30.7)	22 (30.1)	
Low-Middle	228 (21.5)	79 (24.5)	132 (19.8)	17 (23.3)	
Middle	159 (15.0)	49 (15.2)	100 (15.0)	10 (13.7)	
Middle-High	174 (16.4)	49 (15.2)	111 (16.6)	14 (19.2)	
Highest	171 (16.1)	42 (13.0)	119 (17.8)	10 (13.7)	
Relationship Status, No. (%)					χ^2^ (4) = 6.98, p = 0.10^d^
Single	731 (69.0)	208 (64.8)	468 (70.4)	55 (76.4)	
Married/Partnered	300 (28.4)	103 (32.1)	183 (27.5)	14 (19.4)	
Widowed/Divorced/Separated	27 (2.6)	10 (3.1)	14 (2.1)	3 (4.2)	
Have Children, No. (%)	209 (19.9)	90 (28.1) **[B]**	106 (16.0) **[A]**	13 (18.1)	χ^2^ (2) = 19.96, **p < 0.001**^ g^
**Medical Characteristics**					
Cancer type, No. (%)					χ^2^ (14) = 15.56, p = 0.34
Leukemia	377 (35.5)	122 (37.9)	234 (35.1)	21 (28.8)	
Lymphoma	235 (22.1)	69 (21.4)	151 (22.6)	15 (20.6)	
Brain and other nervous system	162 (15.2)	35 (10.9)	111 (16.6)	16 (21.9)	
Endocrine system	59 (5.6)	25 (7.8)	31 (4.7)	3 (4.1)	
Bone and joints	55 (5.2)	18 (5.6)	33 (5.0)	4 (5.5)	
Melanoma	37 (3.5)	13 (4.0)	22 (3.3)	2 (2.7)	
Reproductive	53 (5.0)	16 (5.0)	34 (5.1)	3 (4.1)	
Other	84 (7.9)	24 (7.4)	51 (7.6)	9 (12.3)	
Relapse/Recurrence, No. (%)	139 (13.0)	38 (12.0)	86 (13.1)	14 (19.7)	χ^2^ (2) = 3.08, p = 0.22
Treatment Intensity, No. (%)					χ^2^ (6) = 5.12, p = 0.55^d^
Least intensive	64 (6.0)	25 (7.8)	36 (5.4)	3 (4.1)	
Moderately intensive	332 (31.3)	92 (28.6)	220 (33.0)	20 (27.4)	
Very intensive	523 (49.2)	163 (50.6)	322 (48.3)	38 (52.1)	
Most intensive	143 (13.5)	42 (13.0)	89 (13.3)	12 (16.4)	
**Sleep Characteristics**					
Average sleep, hours					–
Mean (*SD*)	7.3 (1.4)	5.9 (0.6)	7.7 (0.7)	10.6 (1.0)	
Median	7	6	8	10	
Range	4–14	4–6.5	7–9	9.5–14	
Restless sleep (past week), *M* (*SD*)	0.95 (0.98)	1.24 (0.98) **[B]**	0.79 (0.94) **[A, C]**	1.10 (1.01) **[B]**	*F*(2, 1045) = 24.04, **p < 0.001**^ h^

(A) All pairwise comparisons were conducted using the Bonferroni posthoc test. Recommended sleep was the reference group for all pairwise comparisons. (B) Column comparison symbols: A,B, C; bolded letters within a column indicate the significantly different group. Absence of a letter indicates no significant differences. *SES* = socioeconomic status. ^a^Pairwise comparisons not significant; short vs. recommended:* p* = 0.23; long vs. recommended: *p* = 0.48. ^b^Short vs. recommended: *p* = 0.001; long vs. recommended: *p* = 0.13. ^c^Short sleepers were more likely to be employed full- or part-time and long sleepers were more likely to be unemployed. ^d^Fisher’s exact *p*-value. ^e^Long sleepers were less likely to have a college degree. ^f^ Neighborhood SES is a multi-component, census-based composite score [31, 32]. ^g^Short sleepers were more likely to have children. ^h^Short vs. recommended: p < 0.001; long vs. recommended:* p *= 0.032

### Demographic and clinical correlates of short and long sleep duration

Compared to recommended sleep duration, participants with short sleep duration were, on average, older at survey completion (mean age = 27.2 relative to mean age = 25.9,* p* = 0.001) and more likely to be employed full-or-part time (73.2% relative to 66.3%, *p* < 0.001) and have children (28.1% relative to 16%, *p* < 0.001). Compared to recommended sleep duration, participants with long sleep duration were more likely to be unemployed (59.2% relative to 33.7%, *p* < 0.001) and have lower educational attainment (11.0% reported a college degree relative to 31.0%, *p* = 0.001). More restless sleep within the past week was associated with both short (*M* = 1.24) and long (*M* = 1.10) sleep duration, relative to recommended sleep duration (*M* = 0.79; *p*s < 0.032).

### Associations with mental and physical health

Means and standard deviations are shown in Table 2; coefficients, standard errors, and 95% confidence intervals (CIs) are shown in Table 3. Both short and long sleep duration, relative to recommended sleep duration, were associated with lower well-being (*b* = −3.92 and *b* = −6.66, respectively, *p*s ≤ 0.001), higher depressive symptoms (*b* = 1.95 and *b* = 4.15, respectively, *p*s ≤ 0.001), worse self-rated general health (*b* = −0.17,* p* = 0.012 and *b* = −0.55, *p* < 0.001, respectively), and greater log odds of having more comorbid conditions (*b* = 0.33 and *b* = 0.64, respectively, *p*s = 0.001). When controlling for depressive symptoms, the association between short sleep duration and self-rated general health was no longer significant (*p* = 0.24). Relationships between long sleep duration and self-rated physical health (*p* = 0.009) and both short and long sleep duration and comorbid conditions (*p* = 0.031 and *p* = 0.028, respectively) retained significance.

**Table 2:** Means and standard deviations of primary outcomes by sleep duration

	**Total** (*N* = 1,062)			**Short Sleep** **(< 7 h)** (*n* = 322)			**Recommended Sleep** **(7–9 h)** (*n* = 667)			**Long Sleep** **(> 9 h)** (*n* = 73)		
	***n***	***M***	***SD***	***n***	***M***	***SD***	***n***	***M***	***SD***	***n***	***M***	***SD***
Well-being^a^	1,006	47.38	14.96	306	44.95	14.47	634	49.09	14.66	66	42.26	17.30
Depressive Symptoms^b^	1,007	8.50	7.57	307	9.61	8.04	631	7.57	6.92	69	12.01	9.28
Self-rated General Health^c^	1,043	2.35	1.01	317	2.23	0.98	654	2.46	0.99	72	1.89	1.01
Comorbid Conditions^d^	1,062	0.75	1.23	322	0.89	1.37	667	0.62	1.05	73	1.22	1.84

*n*, sample size; *M*, mean; *SD*, Standard deviation. ^a^Mental Health Continuum-Short Form (MHC-SF). ^b^ 13 items from the Center for Epidemiological Studies Depression Scale (CES-D), with the Somatic Subscale excluded. ^c^ 1-item from the 36-item Short Form Survey (SF-36). ^d^ 11-item checklist of comorbidities

**Table 3:** Multivariable associations between sleep duration and health outcomes

Psychosocial Health	Well-being				Depressive Symptoms			
	b	SE	p	95% CI	b	SE	p	95% CI
Sleep Duration								
Recommended	(referent)	–	–	–	(referent)	–	–	–
Short Sleep	−3.92	1.04	< 0.001	−5.96, −1.89	1.95	0.52	< 0.001	0.92, 2.98
Long Sleep	−6.66	1.92	0.001	−10.42, −2.89	4.15	0.95	< 0.001	2.28, 6.02
Age at survey completion	−0.10	0.13	0.43	−0.35, 0.15	−0.01	0.06	0.974	−0.13, 0.12
Female (vs. Male)	1.88	0.93	0.044	0.05, 3.72	0.22	0.47	0.641	−0.71, 1.15
Race and Ethnicity								
Non-Hispanic white	(referent)	–	–	–	(referent)	–	–	–
Hispanic	−2.80	1.06	0.009	−4.88, −0.71	1.28	0.54	0.017	0.23, 2.33
Non-Hispanic Asian	−3.68	1.71	0.031	−7.03, −0.33	1.83	0.85	0.032	0.16, 3.51
Non-Hispanic Black	−3.63	2.26	0.108	−8.07, 0.80	2.71	1.15	0.019	0.44, 4.97
Other or unknown	−3.14	4.54	0.49	−12.06, 5.78	2.43	2.20	0.270	−1.89, 6.75
Age at diagnosis	0.16	0.11	0.175	−0.07, 0.38	−0.04	0.05	0.441	−0.16, 0.07
Treatment Intensity								
Least	(referent)	–	–	–	(referent)	–	–	–
Moderate	−1.83	2.14	0.391	−6.03, 2.36	0.81	1.07	0.451	−1.29, 2.91
Very Intensive	−1.99	2.05	0.334	−6.02, 2.04	0.73	1.03	0.477	−1.29, 2.75
Most Intensive	−3.66	2.32	0.115	−8.21, 0.89	1.01	1.17	0.386	−1.28, 3.31
**Physical Health**	**Self-Rated General Health**				**Comorbid Conditions**			
	b	SE	p	95% CI	b	SE	p	95% CI
Sleep Duration								
Recommended	(referent)	–	–	–	(referent)	–	–	–
Short Sleep	−0.17	0.07	0.012	−0.30, −0.04	0.36	0.11	0.001	0.14, 0.58
Long Sleep	−0.55	0.12	< 0.001	−0.78, −0.31	0.64	0.19	0.001	0.27, 1.00
Age at survey completion	−0.03	0.01	< 0.001	−0.05. −0.02	0.03	0.01	0.043	0.01, 0.05
Female (vs. Male)	−0.02	0.06	0.733	−0.14, 0.10	0.17	0.10	0.098	−0.03, 0.37
Race and Ethnicity								
Non-Hispanic white	(referent)	–	–	–	(referent)	–	–	–
Hispanic	−0.38	0.07	< 0.001	−0.52, −0.25	0.02	0.11	0.875	−0.21, 0.24
Non-Hispanic Asian	−0.18	0.11	0.104	−0.39, 0.04	−0.29	0.20	0.144	−0.68, 0.10
Non-Hispanic Black	−0.34	0.15	0.020	−0.63, −0.05	0.20	0.23	0.390	−0.25, 0.65
Other or unknown	−0.65	0.29	0.023	−1.21, −0.09	−1.14	0.68	0.095	−2.48, 0.20
Age at diagnosis	0.01	0.01	0.158	−0.01, 0.02	−0.02	0.01	0.181	−0.04, 0.01
Treatment Intensity								
Least	(referent)	–	–	–	(referent)	–	–	–
Moderate	−0.13	0.14	0.353	−0.40, 0.14	0.38	0.27	0.169	−0.16, 0.92
Very Intensive	−0.23	0.13	0.081	−0.49, 0.03	0.76	0.26	0.004	0.25, 1.27
Most Intensive	−0.44	0.15	0.004	−0.73, −0.14	1.21	0.28	< 0.001	0.66, 1.76

*SE*, standard error; *CI*, confidence interval. Models adjusted for biological sex, age at study participation, age at diagnosis, race/ethnicity, and treatment intensity

### Moderation by Hispanic ethnicity

Adding the interaction term between sleep duration and Hispanic ethnicity (vs. non-Hispanic white) indicated no evidence of moderation [well-being (short sleep: *b* = 1.73, *p* = 0.46; long sleep: *b* = 2.46, *p* = 0.59), depressive symptoms (short sleep: *b* = 1.00, *p* = 0.51; long sleep: *b* = −3.90, *p* = 0.17), self-rated general health (short sleep: *b* = −0.07, *p* = 0.65; long sleep: *b* = 0.49, *p* = 0.08), and comorbid conditions (short sleep: *b* = −0.28, *p* = 0.27; long sleep: *b* = −0.02, *p* = 0.96)].

## Discussion

The purpose of this study was to characterize sleep duration—an understudied health behavior—in a diverse, population-based sample of young adult CCS, identify demographic and medical correlates, and examine relationships between sleep duration and mental and physical health outcomes. Overall, 30% reported short sleep duration, and 7% reported long sleep. Relative to recommended sleep duration, short sleep was associated with older age, being employed, and having children, and long sleep was associated with being unemployed and lower educational attainment. Notably, we observed no differences in short or long sleep duration by race/ethnicity. Both short and long sleep were associated with worse psychological and physical health compared to recommended sleep duration. Associations with poor physical health remained largely significant when controlling for depressive symptoms (somatic subscale excluded), with the association between short sleep duration and self-rated general health being the only exception. Post hoc analyses did not indicate moderating effects by Hispanic ethnicity (vs. non-Hispanic white). This is the first study to examine the prevalence and correlates of sleep duration in a population-based, ethnically diverse (51% Hispanic) cohort of young adult CCS (aged 19 to 39 at data collection). These findings are clinically important as sleep duration is a modifiable risk factor for improving health among CCS and is amenable to intervention [38].

Encouragingly, many participants reported sleeping the recommended 7 to 9 hours each night (63%). The prevalence of short sleep was lower in our sample of *young adult* CCS (average age at study completion was 26 years) than in previously published data among *adult* CCS (average age at study completion was 34 years)—which ranged from 35.6 to 44% [21, 22]—but greater than estimates among young adults without a history of cancer (21%) [29]. The former finding is to be expected, as older age is associated with shorter sleep duration in both our sample and a previous study of adult CCS [21]. That participants experienced a higher percentage of short sleep than population norms suggests that deficits in healthy sleep among CCS occur early in adulthood, likely due to several factors. Treatment-induced late effects, including pain, cognitive complaints, and comorbid conditions, may interfere with sleep and contribute to shorter sleep duration [39, 40]. Side effects can also catalyze deficits in daytime functioning, which may make it harder to keep up with work or school tasks [41, 42], potentially contributing to shortened sleep duration. More research is needed to establish this link. Long sleep duration, on the other hand, was reported by more participants in our study (7%) than in a previous study of CCS (1.5%) [21], and the prevalence was comparable to two studies looking at long sleep in non-cancer-affected young adults, which ranged from 6–9% [29, 30].

Analyses revealed unique correlates of short and long sleep duration. Older participants were more likely to report short sleep—consistent with a previously published study of CCS [21]—as were those who were employed full- or part-time and had children. Young adulthood is a rich developmental period where competing demands (e.g., school, work, childrearing) may interfere with one’s ability or desire to engage in health-promoting behaviors, including getting adequate sleep. Indeed, these competing demands also partially explain low participation and adherence to psychosocial interventions [43]. Conversely, unemployment and lower educational attainment were associated with long sleep. Long sleep duration may indicate underlying mental and physical health concerns [37] (supported by our analyses), which may impede educational and occupational attainment [44]. Though our analyses focused on demographic and medical correlates, it is worth noting that persistent physical symptoms (as mentioned above) and chronic health conditions may also contribute to both short and long sleep. Literature from non-cancer-affected adults suggests that associations between physical symptoms (e.g., pain and fatigue) and sleep duration follow a U-shaped pattern, with higher symptom levels associated with both short and long sleep [e.g., 45]. Further research on physical symptoms and sleep duration in CCS is warranted, as sleep health is integral to symptom management [12, 13].

We did not observe differences in sleep duration by race/ethnicity or by medical characteristics. In a previous study, Lubas et al. (2021) found that Non-Hispanic white CCS were more likely to report recommended sleep duration, but due to a small number of racial and ethnic minorities, additional differences could not be determined. Among large studies with non-cancer affected adults, higher prevalence of short and long sleep duration has been reported among Latinos/Hispanics [8, 9], Non-Hispanic Blacks [8, 9, 46, 47], and Asians [46, 47], when compared to Non-Hispanic whites. Results, however, are not always consistent and may vary by heritage subgroup, particularly for Latinos/Hispanics (e.g., [8, 9]). Here, we observed comparable percentages among racial/ethnic groups. The findings for Asian and Non-Hispanic Black CCS should be interpreted cautiously, however, due to small sample sizes, and warrant assessment in larger samples. Additional analyses examining relationships among heritage, acculturation, and sleep duration among Hispanic CCS are also warranted, as these may be important modifying factors. We saw no difference in sleep duration across medical characteristics (i.e., cancer type, age at diagnosis, treatment intensity), likely because most of the sample was in long-term survivorship—84% were 10 or more years post-diagnosis.

Compared with recommended sleep duration, both short and long sleep duration were associated with worse mental and physical health. This is consistent with previous research on short sleep duration among non-cancer-affected young adults [29, 48, 49] and CCS [21, 22], but is the first to replicate these findings in a majority-Hispanic sample of young adult CCS. This is also the first study, to our knowledge, to examine associations between long sleep duration and health among CCS. Associations between long sleep duration and poor mental and physical health among *non-cancer-affected* young adults have been reported, but findings are inconsistent [30, 48–50]. For example, Steptoe et al. (2016) demonstrated negative effects of short, but not long, sleep on self-rated general health among 17,645 non-cancer-affected young adults [51]. Results from the current study highlight the relationship between both extremes—short and long sleep duration—and overall health. Associations were particularly strong when comparing short and long sleep duration to recommended sleep duration for diminished well-being, increased depressive symptoms (excluding somatic symptoms), and comorbidities. Relationships between long sleep duration and self-rated physical health and both short and long sleep duration and comorbid conditions retained significance when controlling for depressive symptoms. In post hoc analyses, we observed no moderated effects for Hispanic ethnicity, despite this group reporting lower well-being, greater depressive symptoms, and poorer self-rated overall health (vs. non-Hispanic white). Future research should consider additional modifying factors, including heritage and acculturation (as mentioned previously), as well as other sleep-related domains, such as sleep efficiency, to understand these relationships better. In sum, we observed adverse relationships between both short and long sleep duration, compared to recommended sleep duration, on mental and physical health—even after controlling for relevant demographic, medical, and psychological characteristics—and no moderating effect of Hispanic ethnicity (vs. non-Hispanic white).

Sleep duration influences health in several ways [52]. Limited sleep may lead to an increase in systemic inflammation [5, 6]—which has been linked to greater depressive symptoms [53]; physical symptoms (e.g., fatigue) [54]; and the development of chronic health conditions [55]—and alterations in the autonomic nervous system, which may confer risk for cardiovascular disease [7]. Furthermore, inadequate sleep can interfere with cognitive processes integral to healthy psychosocial functioning, including emotion regulation [56]. Pathways linking long sleep duration to mental and physical health outcomes are less clear [57, 58], though systemic inflammation and metabolic imbalance may be potential biological mechanisms [59]. Long sleep duration may also indirectly lead to poor health behaviors (e.g., more sedentary behavior). Those who are experiencing poor mental and physical health, for example, may be more inclined to sleep for extended periods of time. Indeed, the relationships between sleep duration and mental and physical health are bidirectional. Though our study does not highlight potential mechanisms, elucidating their influence will be an important next step toward understanding why we observed adverse relationships between short and long sleep duration and mental and physical health.

Our findings suggest that many CCS may benefit from sleep assessment and intervention. Approximately one-third of the sample reported sleep duration deficits, and 25% reported sleeping 7 to 7.5 hours each night, just above the lowest recommended threshold. Medical providers play a critical role in identifying those at risk for sleep issues in survivorship and providing referrals; thus, assessing sleep disturbances at survivorship care visits is highly recommended. Indeed, research suggests clinically significant sleep impairment in CCS is often missed or underreported at these visits [60]. Provision of sleep interventions, either by care providers during treatment or survivorship care clinics, is also warranted. Existing interventions for sleep disturbances—including the gold-standard treatment, Cognitive Behavioral Therapy for Insomnia (CBT-I)—have shown promise in improving both sleep and non-sleep outcomes in survivors of adult-onset cancers [61]. However, more research is needed among CCS. To date, only two CBT-I trials (both conducted online) have been conducted with CCS [62, 63]. Results suggest benefits in sleep quality and fatigue, as well as challenges to both recruitment and retention. Young adult CCS may also benefit from less intensive interventions, such as sleep extension [38]. Finally, minoritized populations may experience unique challenges to getting adequate sleep (e.g., acculturation [64]), and the development of culturally tailored interventions is critical. Indeed, emerging evidence suggests greater retention among minoritized populations in culturally tailored sleep interventions [65].

The large, diverse (51% Hispanic) population-based sample of young adult CCS is a notable strength of this study. This is also the first study to examine associations between long sleep duration and health among CCS. However, there are several limitations. Both sleep duration and quality were self-reported at one timepoint using one-item measures, increasing the risk of recall bias and measurement error. Self-reported physical health data were subject to the same reporting bias. Utilizing multi-item scales and assessing additional sleep- and health-related variables would provide a more comprehensive understanding of sleep disruptions and health among CCS. Future research should consider additional sleep-related domains, including sleep regularity, efficiency, and quality, which are relevant to both mental and physical health. Use of objective measures may also mitigate bias. Due to the cross-sectional design, we could not examine bidirectional relationships among participant characteristics, sleep duration, and health. Understanding how these relationships co-occur over time is an important next step. Finally, this sample is representative of Southern California and may not be generalizable to other geographic regions, which may exhibit variations in both sleep duration and health.

This paper is the first to examine prevalence and correlates of sleep duration in a majority-Hispanic sample of young adult CCS. Further research that more fully characterizes sleep characteristics (e.g., sleep duration, sleep disturbance) is warranted, including longitudinal assessments spanning the cancer continuum (e.g., diagnosis, treatment, early survivorship, long-term survivorship) and assessments in large, diverse samples of CCS. Nevertheless, findings from the current study can inform the development of targeted interventions by highlighting at-risk populations of CCS and providing preliminary data on the associations between short *and* long sleep duration on health outcomes in this underserved population.

## Data Availability

De-identified data from this study are not available in a public archive but will be made available (as allowable according to institutional IRB standards) by emailing the corresponding author. The analytic code used to conduct the analyses presented in this study is not available in a public archive. They may be available by emailing the corresponding author. Materials used to conduct the study are not publicly available but can be obtained by emailing the corresponding author.
